# Biases in BCI experiments: Do we really need to balance stimulus properties across categories?

**DOI:** 10.3389/fncom.2022.900571

**Published:** 2022-11-22

**Authors:** Luca La Fisca, Virginie Vandenbulcke, Erika Wauthia, Aurélie Miceli, Isabelle Simoes Loureiro, Laurence Ris, Laurent Lefebvre, Bernard Gosselin, Cyril R. Pernet

**Affiliations:** ^1^ISIA Lab, Numediart Institute, University of Mons, Mons, Belgium; ^2^Department of Cognitive Psychology and Neuropsychology, Interdisciplinary Research Center in Psychophysiology and Electrophysiology of Cognition, Institute of Health Sciences and Technologies, University of Mons, Mons, Belgium; ^3^Department of Neuroscience, Interdisciplinary Research Center in Psychophysiology and Electrophysiology of Cognition, Institute of Health Sciences and Technologies, University of Mons, Mons, Belgium; ^4^Neurobiology Research Unit, Copenhagen University Hospital, Copenhagen, Denmark

**Keywords:** bias avoidance/control, covariates analysis, parametric design, BCI, natural-manufactured, ERP, EEG

## Abstract

Brain Computer Interfaces (BCIs) consist of an interaction between humans and computers with a specific mean of communication, such as voice, gestures, or even brain signals that are usually recorded by an Electroencephalogram (EEG). To ensure an optimal interaction, the BCI algorithm typically involves the classification of the input signals into predefined task-specific categories. However, a recurrent problem is that the classifier can easily be biased by uncontrolled experimental conditions, namely covariates, that are unbalanced across the categories. This issue led to the current solution of forcing the balance of these covariates across the different categories which is time consuming and drastically decreases the dataset diversity. The purpose of this research is to evaluate the need for this forced balance in BCI experiments involving EEG data. A typical design of neural BCIs involves repeated experimental trials using visual stimuli to trigger the so-called Event-Related Potential (ERP). The classifier is expected to learn spatio-temporal patterns specific to categories rather than patterns related to uncontrolled stimulus properties, such as psycho-linguistic variables (e.g., phoneme number, familiarity, and age of acquisition) and image properties (e.g., contrast, compactness, and homogeneity). The challenges are then to know how biased the decision is, which features affect the classification the most, which part of the signal is impacted, and what is the probability to perform neural categorization *per se*. To address these problems, this research has two main objectives: (1) modeling and quantifying the covariate effects to identify spatio-temporal regions of the EEG allowing maximal classification performance while minimizing the biasing effect, and (2) evaluating the need to balance the covariates across categories when studying brain mechanisms. To solve the modeling problem, we propose using a linear parametric analysis applied to some observable and commonly studied covariates to them. The biasing effect is quantified by comparing the regions highly influenced by the covariates with the regions of high categorical contrast, i.e., parts of the ERP allowing a reliable classification. The need to balance the stimulus's inner properties across categories is evaluated by assessing the separability between category-related and covariate-related evoked responses. The procedure is applied to a visual priming experiment where the images represent items belonging to living or non-living entities. The observed covariates are the commonly controlled psycho-linguistic variables and some visual features of the images. As a result, we identified that the category of the stimulus mostly affects the late evoked response. The covariates, when not modeled, have a biasing effect on the classification, essentially in the early evoked response. This effect increases with the diversity of the dataset and the complexity of the algorithm used. As the effects of both psycho-linguistic variables and image features appear outside of the spatio-temporal regions of significant categorical contrast, the proper selection of the region of interest makes the classification reliable. Having proved that the covariate effects can be separated from the categorical effect, our framework can be further used to isolate the category-dependent evoked response from the rest of the EEG to study neural processes involved when seeing living vs. non-living entities.

## 1. Introduction

The use of BCIs extends to an increasing variety of applications requiring signal processing algorithms to extract more and more information from neural signals. Given its high temporal resolution, EEG arouses much interest in recording brain activity. However, its poor spatial resolution makes it difficult to understand the neural mechanisms from the recorded signals. In this context, many neuroscientists use repeated experimental trials based on well-defined stimuli to trigger the so-called ERP. The categorical contrasts, i.e., the differences between the ERPs of different categories, serve as a basis for hypotheses about the inner mechanisms underlying the studied task. The principle of BCI applications is to classify, in real time, each EEG trial into predefined categories related to a specific task. Nowadays, this classification is increasingly performed by machine learning algorithms that learn to identify regions of high categorical contrast from a set of labeled ERPs. An often-seen problem is the generalization of such a classifier to unseen stimuli from subordinate categories, which relates to ensuring the classifier really learns what differs between the categories of stimulus instead of biases specific to the training dataset. In fact, stimulus properties can co-vary with the categories being studied so that the classification could focus on these uncontrolled properties, called covariates, instead of the neural processes that are meant to be measured, as shown by Rousselet et al. (2007). The issue of characterizing the effect of uncontrolled EEG temporal correlation on classification performance has raised many controversies, cf. the case opposing Li et al. (2021) to Palazzo et al. (2020).

To limit the biasing effect on neural mechanism studies, neuroscientists traditionally balance critical known covariates across experimental conditions to minimize their impact on the averaged timelocked ERP signal (Simoes Loureiro and Lefebvre, 2016a). The latter is commonly used to interpret the mechanisms underlying a studied condition. Many studies aim to quantify the covariate effects on the EEG instead of just limiting them by experimental tricks. Thus, Hauk et al. (2006) proposed using a parametric design evaluating the relationship between the data (e.g., P300 ERP peak) and the covariates (e.g., lexical frequency). This parametric design allows the experimenter to keep tight control of the covariates along a continuous space. Applied by Rousselet et al. (2008) to human face processing, this method has revealed that image-noise phase coherence influences the ERP dynamics in spatio-temporal regions of significant categorical contrast (around N170 ERP peak). Similarly, we propose a regression process to separate the categorical effect from the covariate effects and to evaluate how they influence the ERP signal (cf. Sections 2.5, 2.6). This pipeline is developed using the two-level hierarchical linear modeling method of the LIMO EEG toolbox (Pernet et al., 2011).

As demonstrated by Warrington and Shallice (1984) and Tyler and Moss (1997), significant differences in neural processes appear when seeing living things compared to non-living things. This scientific consensus makes the classification between natural living entities and manufactured objects a good candidate to evaluate the influence of uncontrolled covariates on a known task of high neural contrast. In this research, we use EEG data from a priming experiment (Simoes Loureiro and Lefebvre, 2016a) in which two images (the primer and the target) are shown sequentially to a subject who has to answer a question about the target image. The variations in the properties of both images give the opportunity to study the influence of a wide range of covariates on the ERP of both categories. These covariates are chosen among psycho-linguistic variables (as defined by Alario et al., 2004), image visual features (e.g., contrast, homogeneity, compactness), and primer-target relation as described in Section 2.4.

Through the proposed framework, we aim to characterize how the studied covariates modulate the EEG signal by identifying spatio-temporal regions in the ERP significantly affected by this modulation and evaluating the separability between category-related and covariate-related evoked responses. This solution can be applied (1) by BCI experimenters to identify regions of minimal biasing effect and maximal categorical effect to focus the classification process on and (2) by neuroscientists to extract the part of the EEG that is related to categorical effect only. By doing so, they can study unbiased signals without requiring the balance of the covariate values across categories, leading to a higher diversity in the dataset they can use and a significant speed-up in the design of their experiment.

## 2. Materials and methods

**Figure 1:**
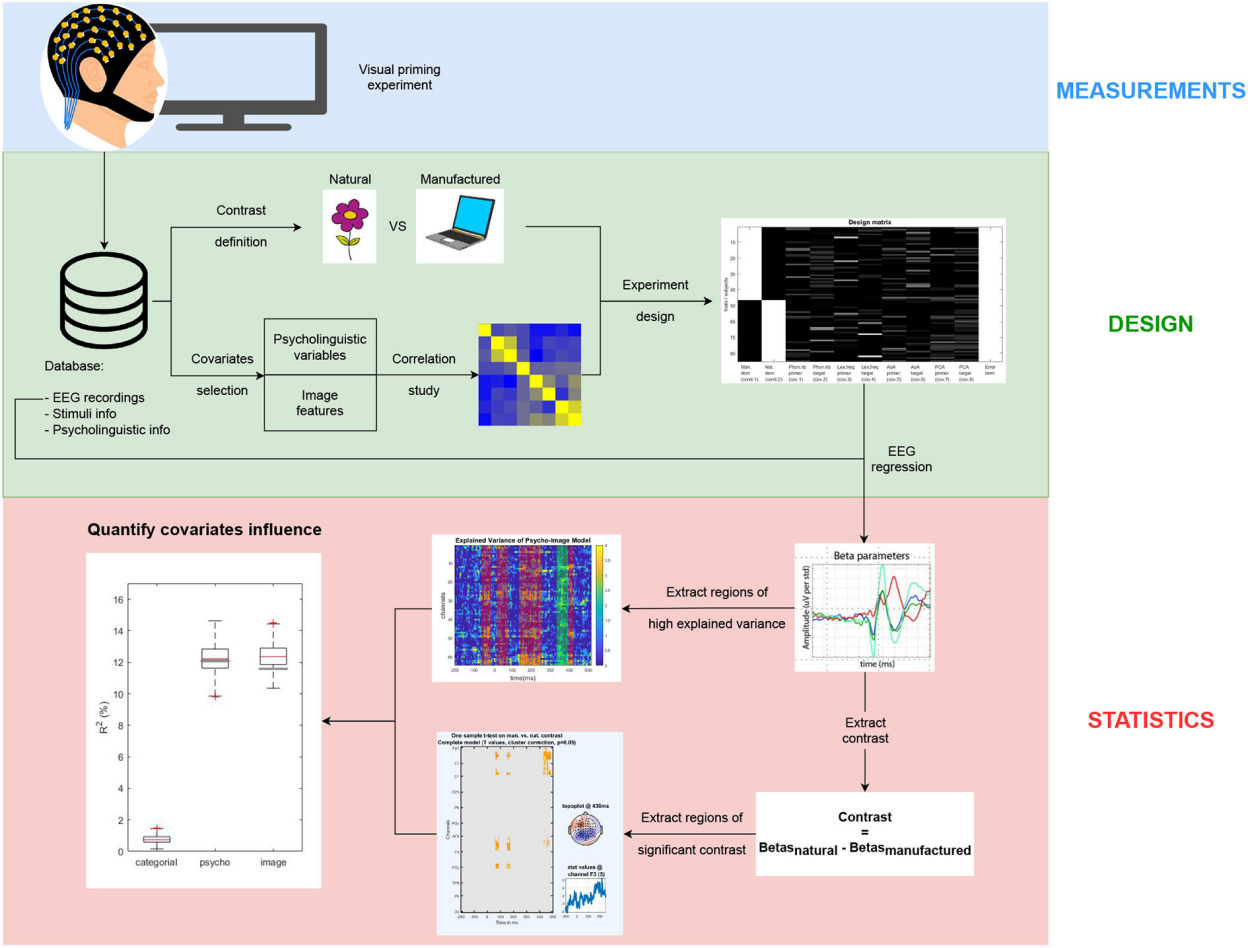
Method overview: (blue) Electroencephalogram (EEG) recordings, (green) design of the model to include all the needed trials information in a standardized way, (red) statistical analysis of the regressed ERP to identify covariates influence.

A detailed description of the proposed method from EEG recordings to statistical analysis is provided, along with freely accessible code at https://github.com/numediart/Covariates_Analysis in order to improve the reproducibility and the extension of this study to other BCI experiments. Figure 1 gives an overview of the full process divided into three main sections: measurements, design, and statistics.

The chosen use case is a priming experiment described in Section 2.2 for which the feature of interest is the target image *a priori* category: natural (i.e., living entity) or manufactured (i.e., non-living object). The selected covariates, whose influence on the distinguishability between both categories on the ERP is studied, are described in Section 2.4. Once the information from the trials is properly formatted, the ERP of each category is regressed subject by subject as described in Section 2.5. Then, the contrast between regression factors (referred to as beta estimates) from both categories is used to extract regions of significant difference between ERP related to manufactured and natural items. By comparing regions of significant categorical contrast and regions where a specific model significantly explains the variance, we can identify which covariates impact the distinguishability between the EEG signals from each category. Finding regions of maximal contrast between categories and minimal covariates influence is highly valuable for the design of future BCI experiments. In addition, a method to separate category-related ERPs from covariate-related ones allows neuroscientists to validate their hypotheses using minimally biased data and speeding-up the design of their experiment. The available code is developed using the open-source FieldTrip (Oostenveld et al., 2010) and LIMO EEG (Pernet et al., 2011) toolboxes.

### 2.1. Participants

Fifteen women and fifteen men healthy right-handed participants (age range 18–35 years old, μ = 24.73, σ = 3.94), who spoke French as a native language with normal or corrected-to-normal vision, were recruited in the central region of Belgium to participate in this study. The sociocultural level was measured according to the highest level of education using the Poitrenaud scale (Hogonot-Diener, 2022) (1 = Elementary School; 2 = Middle School; 3 = High School; 4 = Bachelor Degree; 5 = Master Degree; 6 = Doctoral Degree) resulting in a mean level of 3.8 (σ = 0.4). Handedness was assessed using a French version of the Edinburgh Handedness Inventory (Oldfield, 1971) with all participants being right-handed. Regarding the inclusion criteria, individuals who experienced substance abuse, epilepsy, neurological, and/or psychiatric backgrounds were systematically excluded from the study. All subjects gave their informed written consent after the nature and the potential consequences of the experiment were explained. This study (design and protocol) was approved by the Ethical Board Faculty of Psychology and Education of the University of Mons (Belgium) and was conducted in accordance with the Declaration of Helsinki. The participation was on a voluntary basis without financial compensation.

### 2.2. Stimuli and experimental task

**Figure 2:**
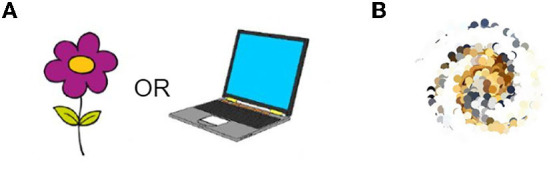
Examples of stimuli for each answer to the task. **(A)** Existing item (correct answer = yes) being either a natural item or a manufactured one, **(B)** Non-existing item (correct answer = no).

The experimental task consists of a semantic priming paradigm, i.e., the participants make a decision about a target picture while ignoring a formerly presented primer picture (Simoes Loureiro and Lefebvre, 2016b). The two images are shown sequentially with the primer being displayed for 90 ms. The requested task was to answer the question: “Is the target picture an existing entity?.” Figure 2 shows examples of stimuli for each expected answer to the task. The primer and target images were selected from a common dataset containing natural entities, manufactured objects (cf. Figure 2A), and abstract shapes, i.e., control stimuli (cf. Figure 2B). The participants answered through two manual press-buttons (yes or no). This experimental design allows multiple effects to be studied by defining categories focused on the items themselves or the relationship between the primer and the target images through the wide object sequence variety of the 431 trials conducted by the subject. Our analysis focuses here on the *a priori* categorical differences on the “existing” target objects only and consequently included 114 trials per subject, each of the studied categories being represented by half of the trials.

### 2.3. Data acquisition, preprocessing, and epoching

Electroencephalogram data were recorded at a sampling rate of 2,048 Hz with a Biosemi Active-Two system from 64 active Ag/AgCl electrode sites, with a Biosemi EEG cap following a 10–20 montage. The Electrooculogram (EOG) was recorded bipolarily from the outer canthi of both eyes and above and below the left eye. The ground electrode was placed on the forehead between Fp1 and Fp2. Electrode impedance was kept below 10*kΩ* through measurements just before and after the experiment.

Segmented EEG trials with minimal noise level and artifacts are required for optimal processing of the data inducing the need for a proper preprocessing pipeline, which code is freely available at https://github.com/numediart/PreprocEEG. The preprocessing steps were performed in the open source FieldTrip software (Oostenveld et al., 2010) following good practice recommendations from the OHBM COBIDAS MEEG committee (Pernet et al., 2020). It is sequenced in eight different steps:

1. Bad channels are removed by visual inspection.
2. Ocular artifact rejection is performed through a multi-channel Wiener filter (Somers et al., 2018) that requires a manual initialization by annotating some artifactual segments. The filter parameters are then automatically tuned to detect the targeted artifacts and remove them while minimizing the degradation of the “true” neural signal. This step is first applied to blinking artifacts and then to eye movements.
3. Detrending, demeaning and low pass filtering (4th order Butterworth, 200 Hz) are applied.
4. Data are epoched as segments starting 500 ms before and ending 1,000 ms after the target onset and downsampled to 512 Hz.
5. The “Zapline” algorithm (de Cheveigné, 2020) is applied on each segment to reduce power line noise using a combination of spectral and spatial filtering.
6. Muscle artifacts are removed using the EEMD-CCA algorithm implemented in the ReMAE toolbox (Chen et al., 2020). Ensemble Empirical Mode Decomposition (EEMD) transforms the signal into intrinsic modes and every mode with an autocorrelation value (lag = 1) below a specific threshold is considered as potential artifactual components (a high threshold is recommended, we use one of 0.9). A Canonical Component Analysis (CCA) is then applied to the potential artifacts to estimate maximally autocorrelated and mutually uncorrelated sources. All sources with autocorrelation values (lag = 1) lower than a chosen threshold (depending on the experimental constraints, we consider T = 0.5 in this experiment) are treated as artifacts and are set to zero. The last step consists of an inverse CCA followed by an inverse EEMD to obtain the cleaned EEG signal (Chen et al., 2019).
7. A baseline correction considering a baseline window between 500 ms before the target onset and 200 ms before the same onset is applied. This corresponds to the period of no stimulation preceding the primer onset. The final retained segments start 200 ms before and end 500 ms after the target onset.
8. The signal is finally re-referenced to the common average of all the electrodes.

### 2.4. Selection of variables

The proposed method aims to identify the effect of uncontrolled variables that are commonly balanced across experimental conditions in EEG experiments due to their high potential biasing effect on the ERP signals. The scope of this study is therefore limited to the selected covariates, without aiming at any extrapolation. The variable selection was done among the psycho-linguistic variables proposed by Alario et al. (2004) as well as image properties considering primer and target items separately (cf. Section 2.2). One additional covariate measured was the visual similarity between primer and target items. The value of this similarity is defined by a Poitrenaud test (Hogonot-Diener, 2022) during the pre-test of the experiment (1 = primer has a totally different shape than target, 5 = the shape of the primer is the same as the target). Table 1 Provides a short description of each covariate (see text footnote ^1^).

**Table 1:** Description of selected covariates with a separation between psycho-linguistic variables and image properties.^1^

**Covariate name**	**Description**
Phoneme number	Number of phonemes in the French name of the item
Lexical frequency	How often the item appear in the literature
Movie frequency	How often the item appear in movies
Age of acquisition	At what age we learn the meaning of the item
Visual complexity	Level of detail or intricacy contained within the image
Familiarity	How often we meet the item in our daily life
Imageability	How easily the item will evoke a clear mental image
Entropy	Minimal number of bits required to encode the image
Contrast	Difference in luminance of the image
Correlation	How correlated neighboring pixels are
Homogeneity	How close pixel values are to the mean pixel value
Energy	Measure of the localized change of the image
Compactness	How closely packed the pixels of the item are
Ratio	Length-width ratio of the item
Number of spectral clusters	The variety of frequencies in the image
High frequency energy	Energy of spectral cluster with the highest frequency
Highest frequency	Centroid of the spectral cluster of highest frequency
Maximum spectral distance	Distance between spectral clusters of lowest and highest frequency
Visual similarity	How similar the primer and the target picture shapes are

**Figure 3:**
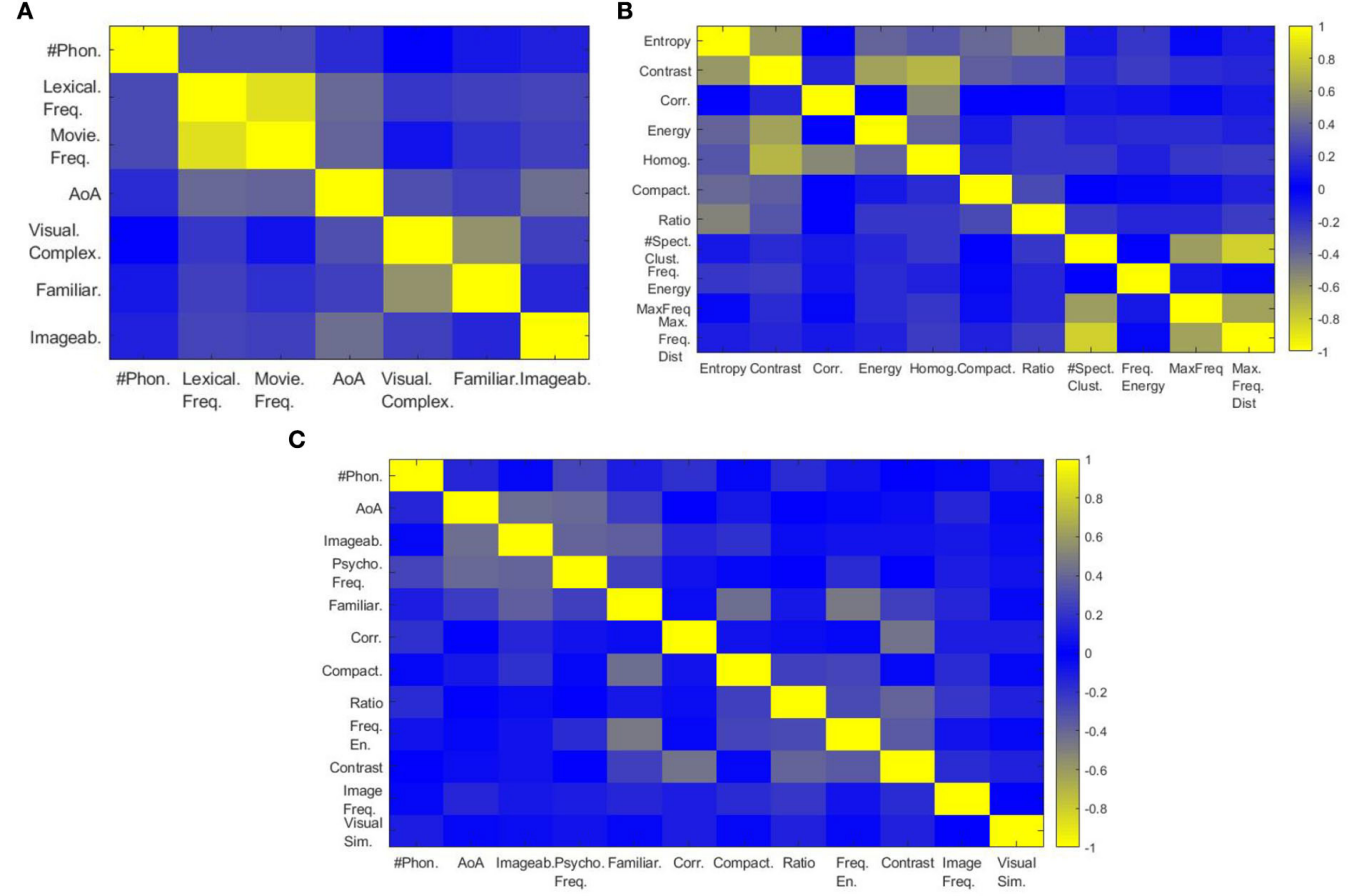
Correlation analysis of covariates. **(A)** correlation between psycho-linguistic variables, **(B)** correlation between image properties, and **(C)** correlation between selected covariates.

Having considered many covariates, we first performed a correlation analysis to select the most useful features in order to minimize the model dimensionality while retaining relevant information. This analysis was performed on psycho-linguistic and image variables independently. In Figure 3A, we can observe that lexical and movie frequencies are highly correlated [correlation factor (called c) = 0.878], we, therefore, performed a Principal Component Analysis (PCA) and kept the first component (explained variance = 93.92%) to represent the common effect. For simplicity, we will call this new variable “psycho frequency” for the rest of the paper. As visual complexity and familiarity were anti-correlated (c = –0.560), a PCA was applied and the first component (explained variance = 87.01%) was defined as “familiarity.” Phoneme number and Age of Acquisition (AoA) were weakly correlated with other covariates and were, thus, kept as such. This analysis allowed us to go from 7 to 5 psycho-linguistic dimensions. In Figure 3B, we see that entropy, contrast, energy, and homogeneity are highly correlated (lowest c = 0.593). The first component of a PCA was chosen to summarize them, reflecting “contrast” (explained variance = 63.76%). The number of spectral clusters, the maximum frequency, and the maximum distance between spectral clusters are highly correlated (lowest c = 0.629) and similarly, we used PCA and kept the first component reflecting “image frequency” (explained variance = 78.62%). Correlation, compactness, and length-width ratio were considered as independent covariates regarding their low correlation score with other features, reducing dimensionality from 9 to 7. Figure 3C summarizes the correlation between the 12 selected covariates after applying dimensionality reduction.

### 2.5. Linear modeling

**Figure 4:**
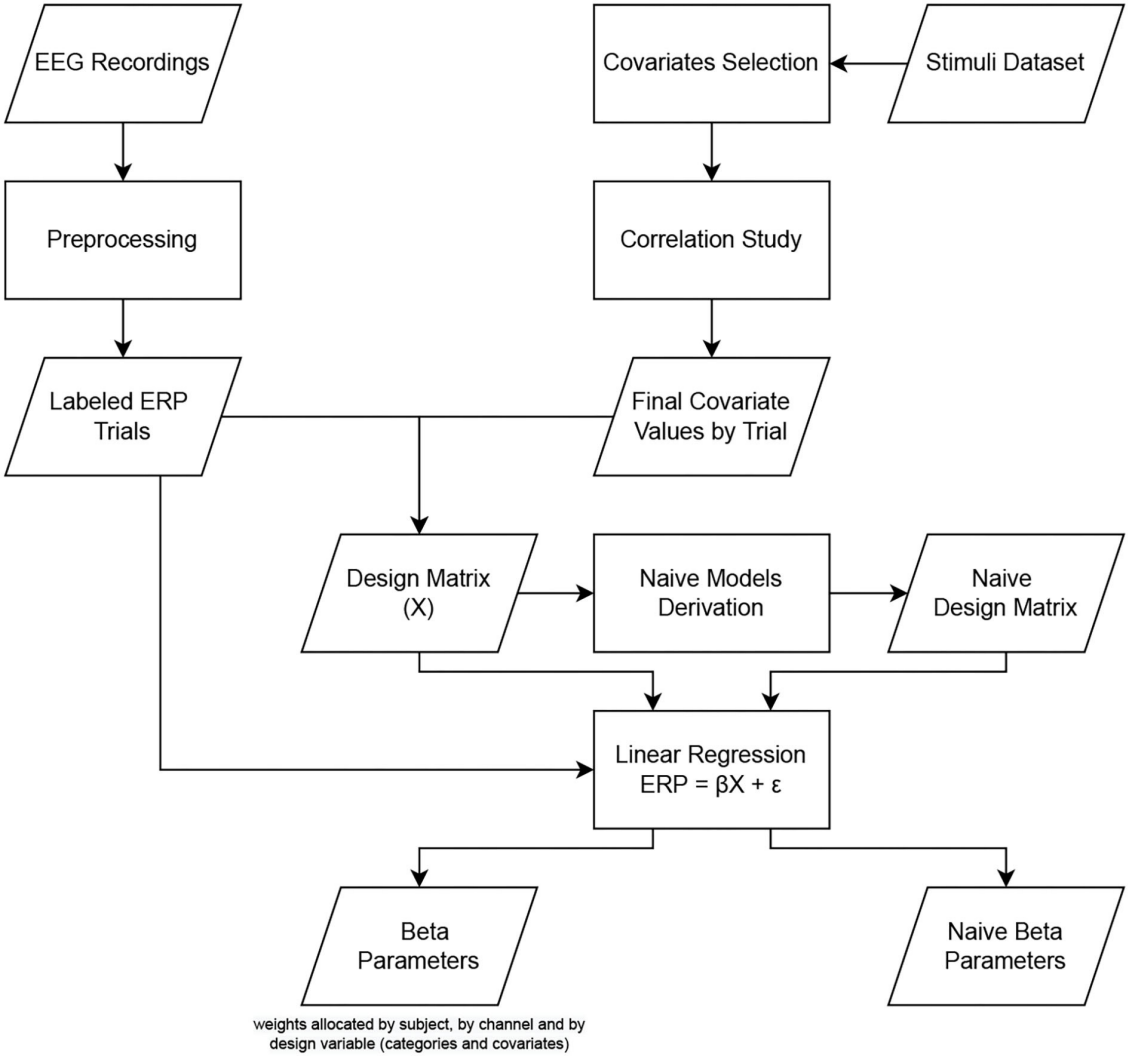
Pipeline used for the first level analysis, i.e., the linear modeling, of the dataset.

**Figure 5:**
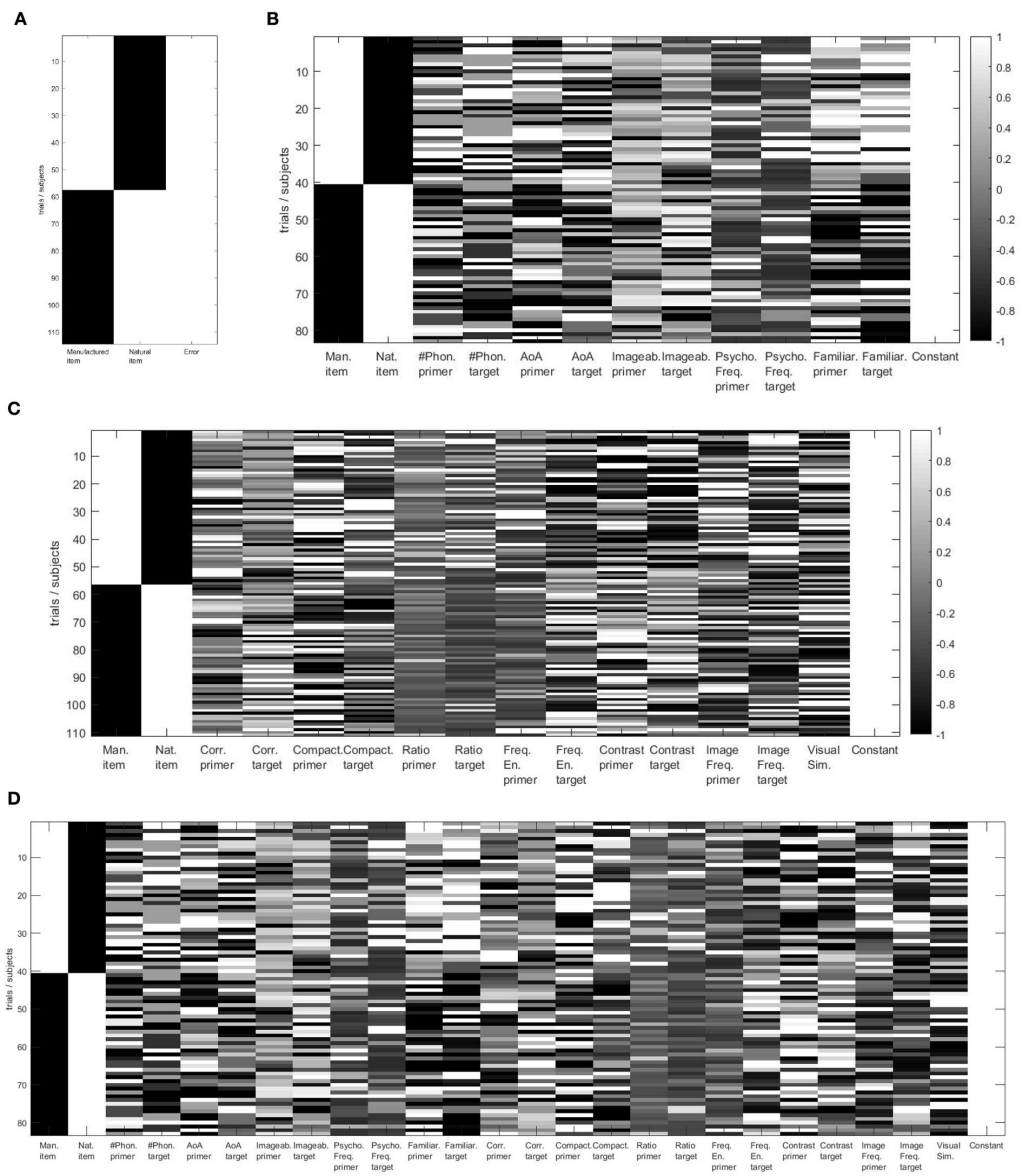
Design matrices. **(A)** Control model (only categories and error terms, 3 dimensions), **(B)** psycho model (13 dimensions), **(C)** image model (16 dimensions), **(D)** psycho-image model (26 dimensions). The two first columns representing the categories are coded as binary values (–1 or 1), while columns corresponding to covariates have continuous values representing the z-score computed through all the trials.

To consider inter-subject variability, the analysis is performed using a 2-level hierarchical process, as proposed by Pernet et al. (2011) in the LIMO EEG toolbox. This hierarchical general linear model consists of a multivariate linear regression divided into 2 levels of analysis. At the first level, the parameters are estimated by the subject at each time point and each electrode separately. Then, at the second level, the parameters estimated at the first level are integrated across subjects to compute robust statistics. The inter-subject variance is, therefore, modeled by the constant term of the regression process at the first level of analysis, while the statistical tests (second level) are performed on the regressed parameters (called beta estimates). This section focuses on the first level analysis that performs linear modeling of the ERP trials as summarized in Figure 4. In this research, we analyzed the effect of psycho-linguistic and image feature covariates on the ERP independently to identify the most critical one, if there is any difference. To do so, we performed the analysis using four different models. As shown in Figure 5, the first model (called “categorical model,” Figure 5A) only considers the categorical variables, the second model (called “psycho model,” Figure 5B) focuses on psycho-linguistic variables, the third one (called “image model,” Figure 5C) only takes into account image features, and the last one (called “psycho-image model,” Figure 5D) encompasses all the covariates. Note that we included the visual similarity in the image model for completeness. The design matrix links each trial with the corresponding category through binary values, the first column representing manufactured items and the second column being related to natural items. The covariates, as continuous variables, are represented by their z-score computed throughout all trials. The regression process aims to obtain an optimal representation of the recorded ERPs for each subject. Depending on the designed model, the beta estimates give the linear combination of categorical variables and covariates that best fits the recorded EEG trials using a General Linear Model (GLM). The regression is done in a parallel way for each subject using the LIMO EEG toolbox through the *limo_batch* function. The computation of beta estimates is presented in Equations (1) and (2) where ERP represents the recorded EEG trials, β the searched parameters, X the design matrix and ϵ the error term. This operation is performed on one channel at a time, fitting all trials simultaneously.


(1) $$\begin{array}{l} ERP=\beta X+\epsilon , \end{array}$$

(2) $$\begin{array}{l} \beta =diag\left({\left(X^{T}X\right)}^{-1}X^{T}ERP\right) \end{array}$$

**Figure 6:**
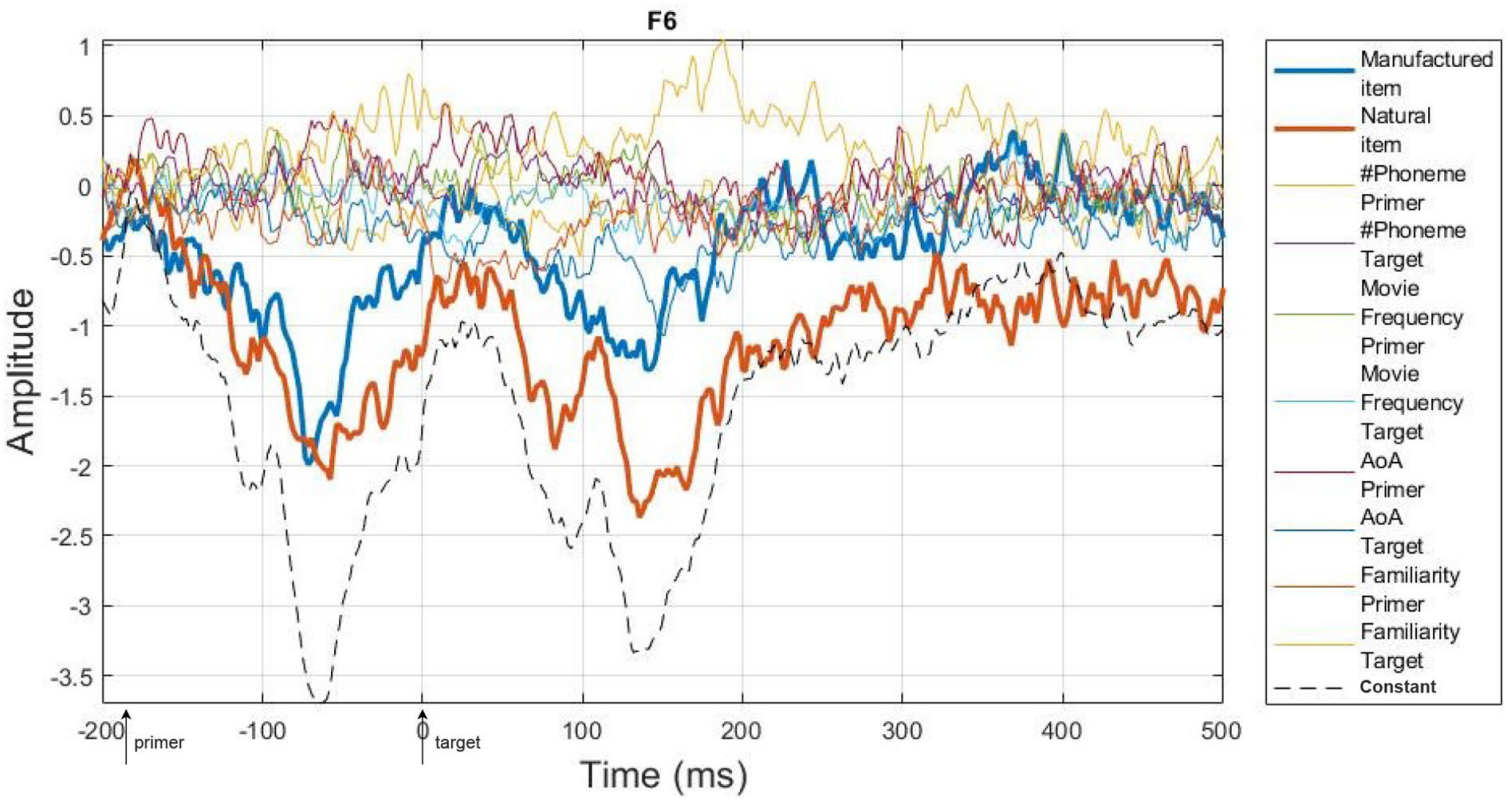
Trimmed mean (20% of trimming) of beta estimates across subjects on F6 electrode using “psycho” model. The two bold lines represent the categorical variables (manufactured and natural items) while the black dashed line belongs to the constant term. All other signals are related to the covariates (cf. legend). The arrows on the x-axis show the appearance of the primer and the target images.

Figure 6 represents the trimmed mean (20% of trimming) of the beta estimates across subjects on one electrode (F6) using the psycho model. This example shows that categorical variables have a larger weight on the regression (higher amplitude of the corresponding beta estimates) than covariates and that the constant term encompasses the general ERP behavior following the appearance of two sequential images.

By computing the difference between beta parameters belonging to each of the categorical variables, we can obtain the categorical contrast effect highlighting the ERP variations that are mainly due to the origin of the presented item, i.e., the effect we want to study. Equation (3) shows the computation of the contrast signal from beta estimates, where c is the contrast. This operation is performed on each subject and each electrode separately.


(3) $$\begin{array}{l} c=\beta_{manufactured}-\beta_{natural}, \end{array}$$

From the contrast parameter, a statistical analysis across subjects can highlight spatio-temporal regions of significant difference between both categories, as described in Section 2.6.

### 2.6. Statistical inference

**Figure 7:**
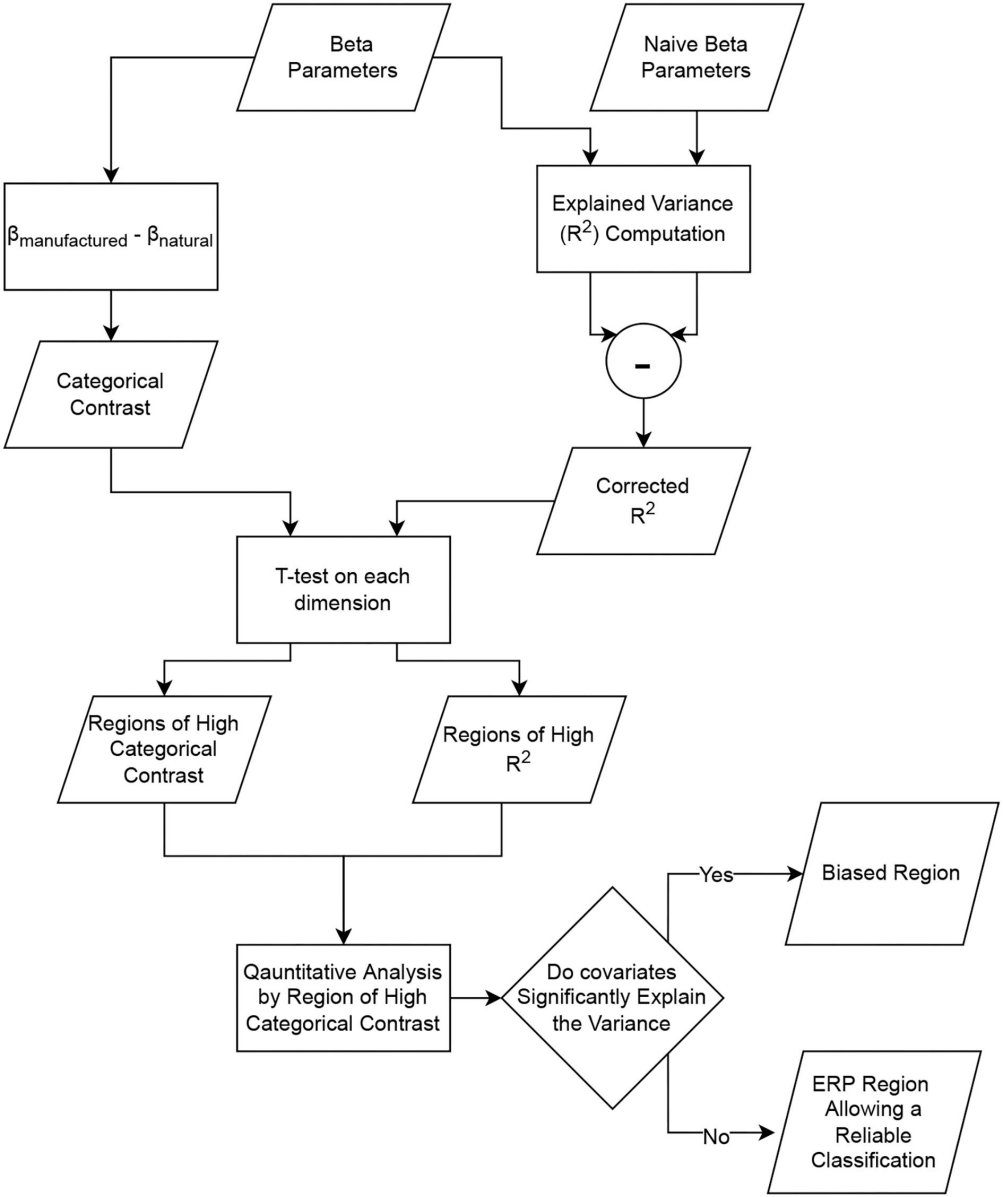
Pipeline allowing the identification of the regions of interest for the classification and the regions prone to covariate bias.

The statistical inference on the regressed signals enables the identification of the spatio-temporal regions of the ERP, allowing a reliable classification between categories and regions prone to covariate bias. The process is summarized in Figure 7.

LIMO EEG proposes tools to perform robust statistics on regressed parameters, such as the Yuen *t*-test (i.e., *t*-test on trimmed mean) alongside bootstrap to account for multiple tests [spatio-temporal clustering and Threshold Free Cluster Enhancement (TFCE)—Pernet et al., 2015] Based on these methods, the second level analysis allows us to identify clusters of significant effect. Highlighting spatio-temporal areas of high categorical contrast is essential to know the regions a BCI algorithm will target to perform the classification task. One-sample *t*-tests were, therefore, run across subjects on the contrast parameters obtained from the categorical model as well as from the psycho-image model, followed by a Multiple Comparison Correction (MCC) using spatio-temporal clustering to identify regions that can be targeted by the classifier. Then, a study of the percentage of the ERP variance that is explained by a model is necessary to reveal the regions where the model properly fits the data. To establish a fair comparison between the explained variances (*R*^2^) of the different models, the effect of the increase of dimensionality must be controlled. For this purpose, we introduced new models (called “naive” models) whose aim is to simulate the effect of changing the model dimensionality. To build a naive model, we use a design matrix on which the two first columns replicate the initial model (to keep the same category for each trial), and the covariate columns are generated as random vectors from a multivariate normal distribution whose mean is zero for every column (as we used the z-score in the initial models) and the covariance matrix has the same rank as the corresponding model. This design matrix is then used to perform the ERP regression as previously described. This process is repeated 30 times with the same categorical design but different random values for each naive model type. The beta estimates corresponding to the categories are averaged over the 30 repetitions to allow the study of the effect of the increase in dimensions on the categorical effect, and the *R*^2^-values are averaged over repetitions to quantify the increase of explained variance that is due to the dimensionality effect. We, therefore, created three naive models corresponding to the psycho, image, and psycho-image models used in the study. The way the explained variances of the different models are combined in the statistical analysis is summarized in **Figure 9** considering the example of the study of the effect of psycho-linguistic variables on the explained variance. The explained variance belonging to a specific model is computed as the difference between the explained variance of the model and that of the corresponding naive model. By applying a one-sample *t*-test to the explained variance across subjects followed by an MCC using spatio-temporal clustering, regions where the covariates influence the way a model fits the ERP can be identified. In fact, as the categorical effect is modeled identically in both the actual and the naive models, the only remaining effect is the influence of the covariates.

### 2.7. Effects separability

**Figure 8:**
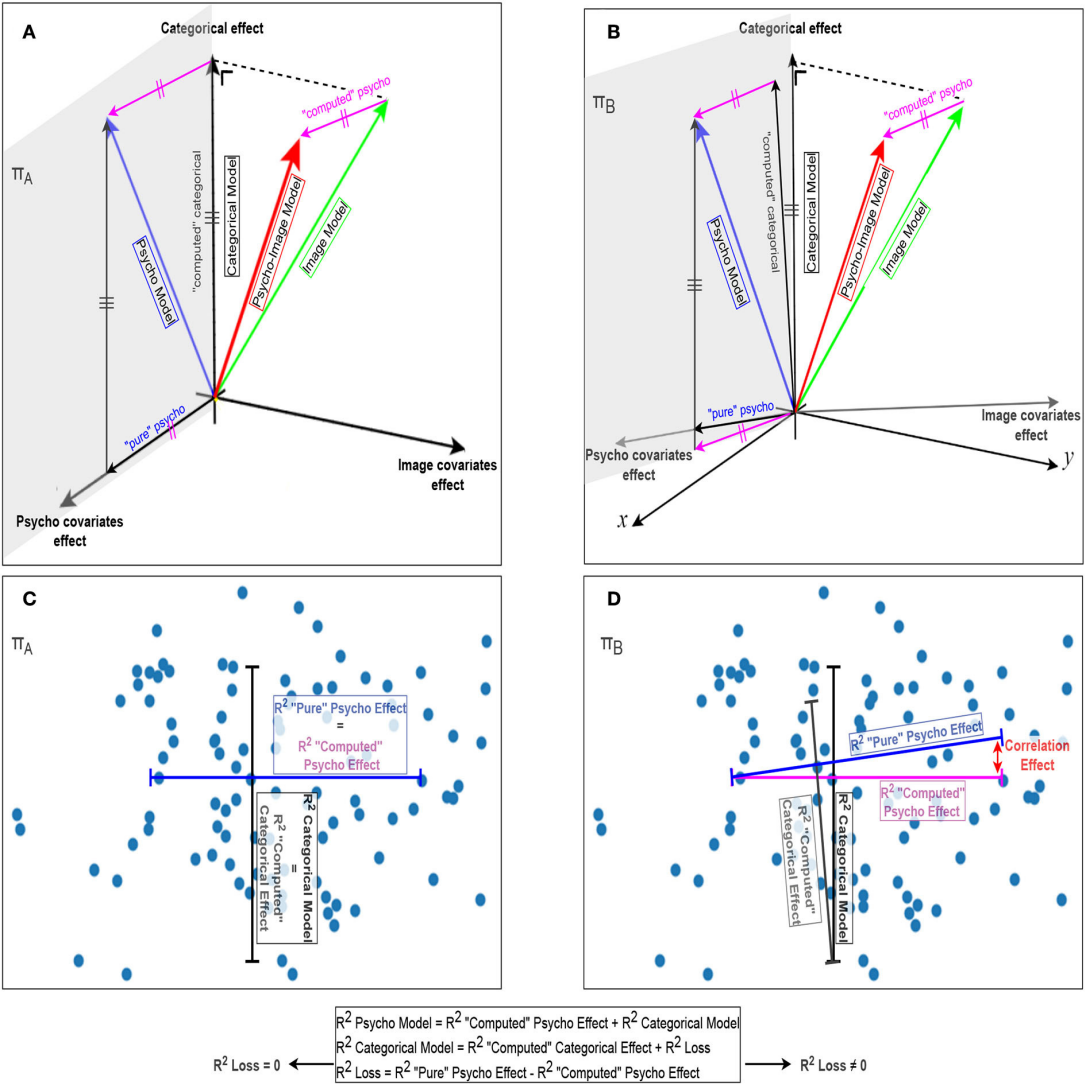
Geometrical representation of the combination of the different models. The left part relates to the ideal situation where the categorical effect, the effect of the psycho covariates, and the image covariates effect are orthogonal to each other, while the right part represents the real-life case of non-orthogonality. **(A,B)** Vectorial representations of the categorical, psycho, image, and psycho-image models and the different effects resulting from their combination, with a focus on the effect of the psycho covariates. **(C,D)** The corresponding projections on the π planes where the *R*^2^-values are computed for a given data set and represented as segments in their corresponding directions. The correlation effect causing the loss in explained variance is represented in red in **(D)**.

**Figure 9:**
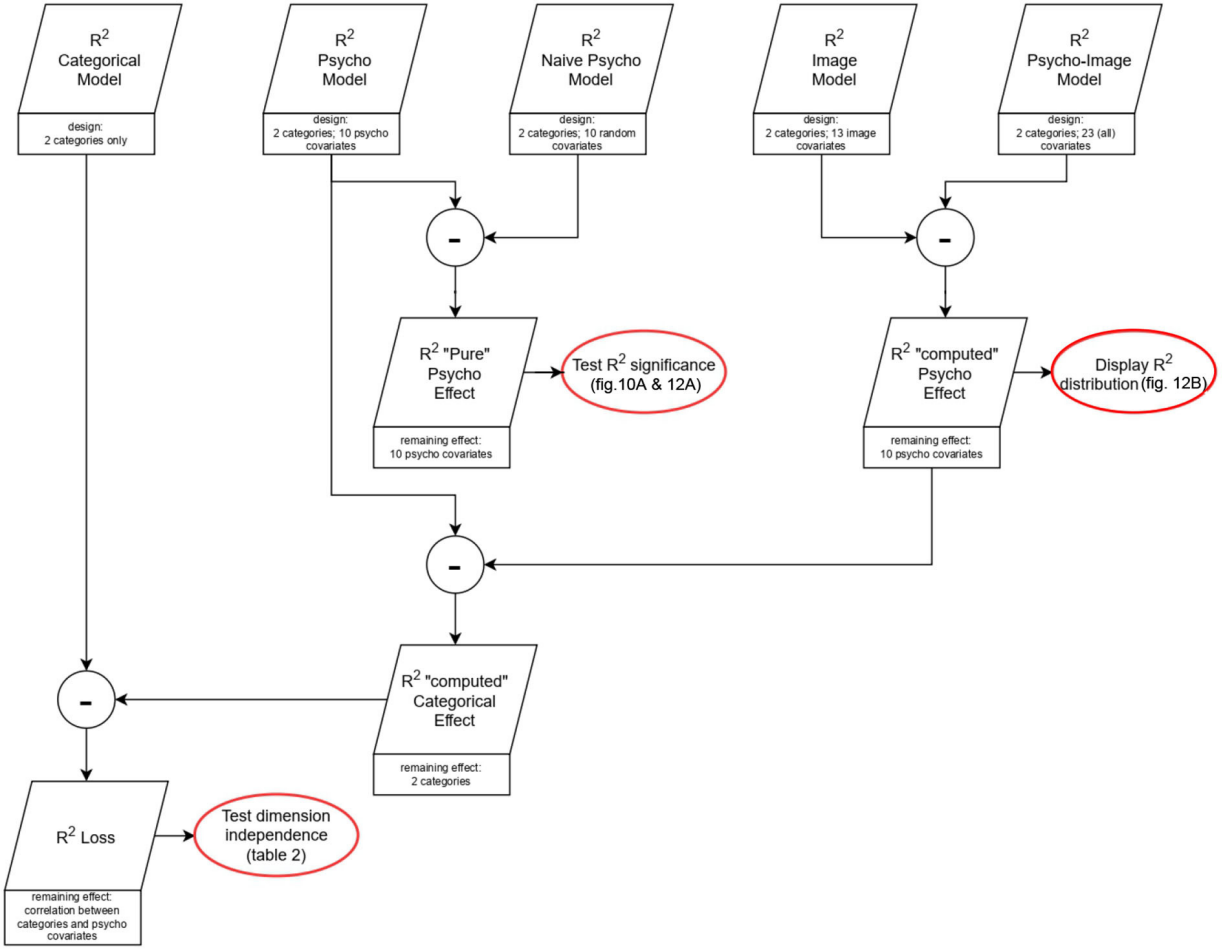
*R*^2^ combination for statistical inference. Example of the study of the effect of psycho-linguistic variables on the explained variance.

To evaluate how separable the biasing covariate effect is from the desired categorical effect, we have to quantify how their potential correlation affects the variance that can be explained by the regressed signals. Figures 8A,B provide a graphical representation of how the different models are combined to extract the contribution of the partial effects required to compute the statistical effects of interest. In Figures 8C,D, a representation of the differences explained variances, as segments in the associated directions, is given. The correlation effect is totally absent in the ideal case of orthogonality, i.e., zero correlation, between the categorical effect, the psycho covariates effect, and the image covariates effect as shown in Figures 8A,C. However, this correlation effect is responsible for a loss in explained variance when considering non-orthogonality between the different effects as Figures 8B,D illustrate. For sake of clarity, we intentionally omitted the dimensionality effect from Figure 8 as adding it would lead to a 4-dimensional problem and would therefore require an additional computation step to obtain the loss in explained variance, as shown in Figure 9. When considering all the dimensions, this loss in explained variance due to the correlation between categorical and covariate effects (*R*^2^ loss) is computed as the difference between the total categorical effect (identified using the categorical model) and the computed categorical effect. The block diagram presented in Figure 9 illustrates that the *R*^2^ loss can be computed using Equations 4a and b where the “computed” psycho effect is the one used to derive the *R*^2^ distribution (cf. **Figure 12B**).


(4a) $$\begin{array}{l} R_{computed\text{\_}categorical\text{\_}effect}^{2}\\ \;=R_{psycho\text{\_}model}^{2}-\left(R_{psycho\text{\_}image\text{\_}model}^{2}-R_{image\text{\_}model}^{2}\right)\\ \;=R_{psycho\text{\_}model}^{2}-R_{computed\text{\_}psycho\text{\_}effect}^{2} \end{array}$$

(4b) $$\begin{array}{l} R_{loss}^{2}=R_{categorical\text{\_}model}^{2}-R_{computed\text{\_}categorical\text{\_}effect}^{2} \end{array}$$

As illustrated in Figures 8A,B, the “computed” psycho effect is obtained by subtracting the image model effect from the psycho-image model effect. This “computed” psycho effect can be considered as part of the effect of the psycho covariates that are not correlated to the categorical effect. Therefore, when subtracting this “computed” psycho effect from the psycho model effect, only the categorical effect remains. This “computed” categorical effect is composed of the actual categorical effect and the part of the psycho covariate effect that is correlated to the categories. The latter component is responsible for the deviation between the categorical model effect and the “computed” categorical effect and can be obtained as the vectorial difference between both. These operations can directly be done on the *R*^2^-values as the explained variance of a joint effect, e.g., psycho model effect that combines categorical and psycho covariate effects is equal to the sum of the explained variances from each of these effects in the ideal case of orthogonality. However, when correlated, this sum is affected by the non-orthogonal part of the considered effects and a loss in *R*^2^ starts to be propagated across the computations.

The separation between the categorical effect and a specific covariate effect is possible if the *R*^2^ loss is significantly lower than the variance explained by the categorical model. This comparison is done within the spatio-temporal cluster of interest using a specific covariates model.

Having proved the separability between categories and covariates, we identify the spatio-temporal regions in the ERP where the categorical effect does not overlap with regions of significant covariate effects. These regions can therefore be used to perform an unbiased classification between the studied categories whatever the balance in the covariate values across those categories.

## 3. Results

**Figure 10:**
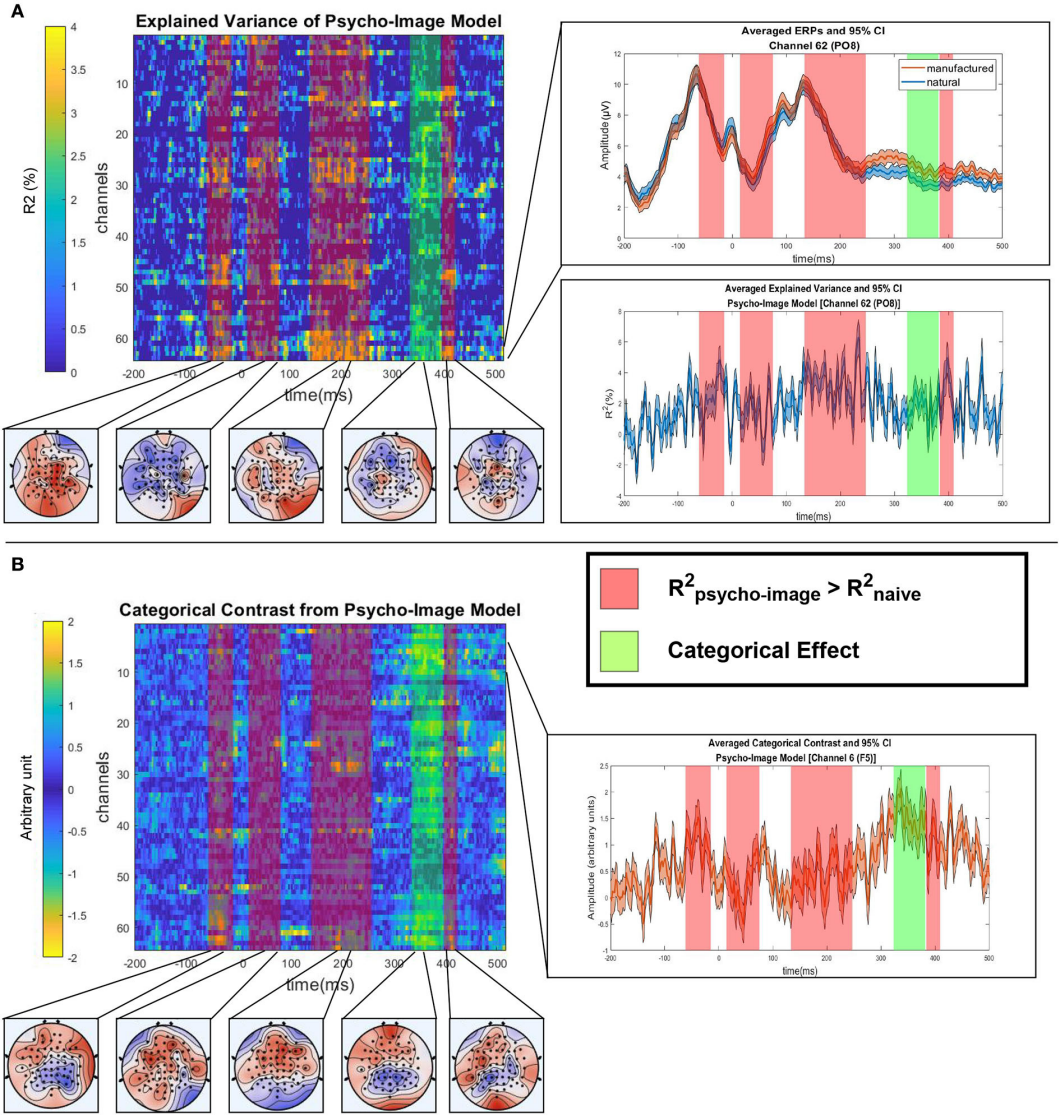
Statistical analysis of the psycho-image model. **(A)** Trimmed mean of the explained variance ($R^{2}-R_{naive}^{2}$) across subjects with corresponding regions of significant explained variance (red bands) and significant categorical contrast (green band). For each highlighted area, the topological view is shown (bottom). On the channel corresponding to the maximum *R*^2^ (PO8 electrode), the averaged ERPs of both categories (top right) and the *R*^2^ timecourse (bottom right) are displayed. **(B)** Trimmed mean of the categorical contrast across subjects with significant regions highlighted and the corresponding topological views (bottom). On the channel corresponding to maximum contrast (F5 electrode), the averaged contrast parameter (β_*man*._−β_*nat*._) is displayed.

As the objective of this study is to reveal the influence of the experimental covariates on the distinguishability of the categorical effect on the EEG, we first ran the statistical analysis described in Section 2.6 on the psycho-image model to extract both categorical and covariate effects when considering all the selected variables. Using this model, we ensure that the identification of significant categorical contrast was not biased by the experimental covariates. Figure 10 shows the explained variance (Figure 10A) and the categorical contrast (Figure 10B) of the psycho-image model along with the 20% trimmed mean ERP. The one-sample *t*-test followed by an MCC using spatio-temporal clustering reveals a cluster of significant categorical contrast from 326 to 371 ms (max *T*-value 5.16 at 334 ms on channel F5, corrected *p* = 0.01) and four clusters of significant *R*^2^: cluster 1 starts at –62 ms and ends at –14 ms (max *T*-value 4.78 at –30.42 ms on channel C2, corrected *p* = 0.03), cluster 2 starts at 14 ms and ends at 75 ms (max *T*-value 4.57 at 68.12 ms on channel PO4, corrected *p* = 0.03), cluster 3 starts at 133 ms and ends at 247 ms (max *T*-value 6.29 at 190.73 ms on channel PO8, corrected *p* = 0.01) and cluster 4 starts at 383 ms and ends at 408 ms (max *T*-value 5.61 at 391.21 ms on channel C1, corrected *p* = 0.02). As the regions where the variance is significantly explained by the values of the covariates do not overlap the cluster of significant categorical contrast, we could assume that the identified categorical effect is not influenced by the chosen covariates when using the psycho-image model.

**Figure 11:**
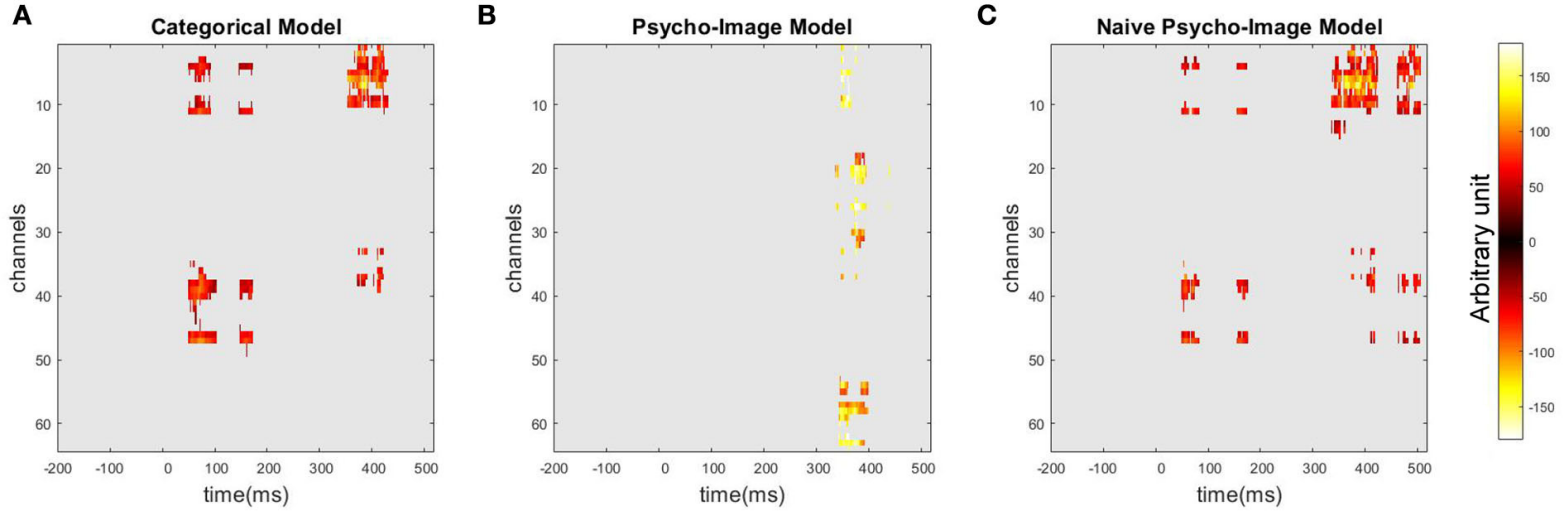
Thresholded maps of the categorical contrast showing spatio-temporal regions of significant categorical contrast using a one-sample *t*-test followed by an MCC with spatio-temporal clustering. These regions are extracted from the categorical model **(A)**, the psycho-image model **(B)**, and the naive psycho-image model **(C)**.

To identify spatio-temporal regions of the ERP that can be wrongly interpreted as clusters of significant categorical contrast if the covariate effects are not modeled, Figure 11 shows the thresholded maps of the categorical contrast obtained when using the categorical model (Figure 11A), the psycho-image model (Figure 11B), and the naive psycho-image model (Figure 11C). We can observe that, on top of the actual region of high contrast between the studied categories, the 3-dimensional categorical model detects two more clusters: one between 43 and 95 ms (max *T*-value 6.37 at 67.14 ms on channel F1, corrected *p* = 0.01), overlapping with the second *R*^2^ cluster of the full psycho-image model, and the other one between 137 and 163 ms (max *T*-value 5.99 at 156.98 ms channel FC1, corrected *p* = 0.04), overlapping with the third *R*^2^ cluster of the psycho-image model. When using the 26-dimensional naive model, similar clusters in excess appear with the first cluster ranging from 43 to 75 ms (max *T*-value 6.01 at 51.51 ms on channel FCz, corrected *p* = 0.02) and the second one from 147 to 167 ms (max *T*-value 4.49 at 160.89 ms on channel FCz, corrected *p* = 0.02), but an additional region between 446 and 489 ms (max *T*-value 5.63 at 483.15 ms on channel F3, corrected *p* = 0.01) is also considered a cluster of significant categorical contrast. These results show that existing biases in the dataset can be badly exploited in the regression process and this biased effect becomes higher as the complexity of the model used increases, as discussed in Section 4.

**Figure 12:**
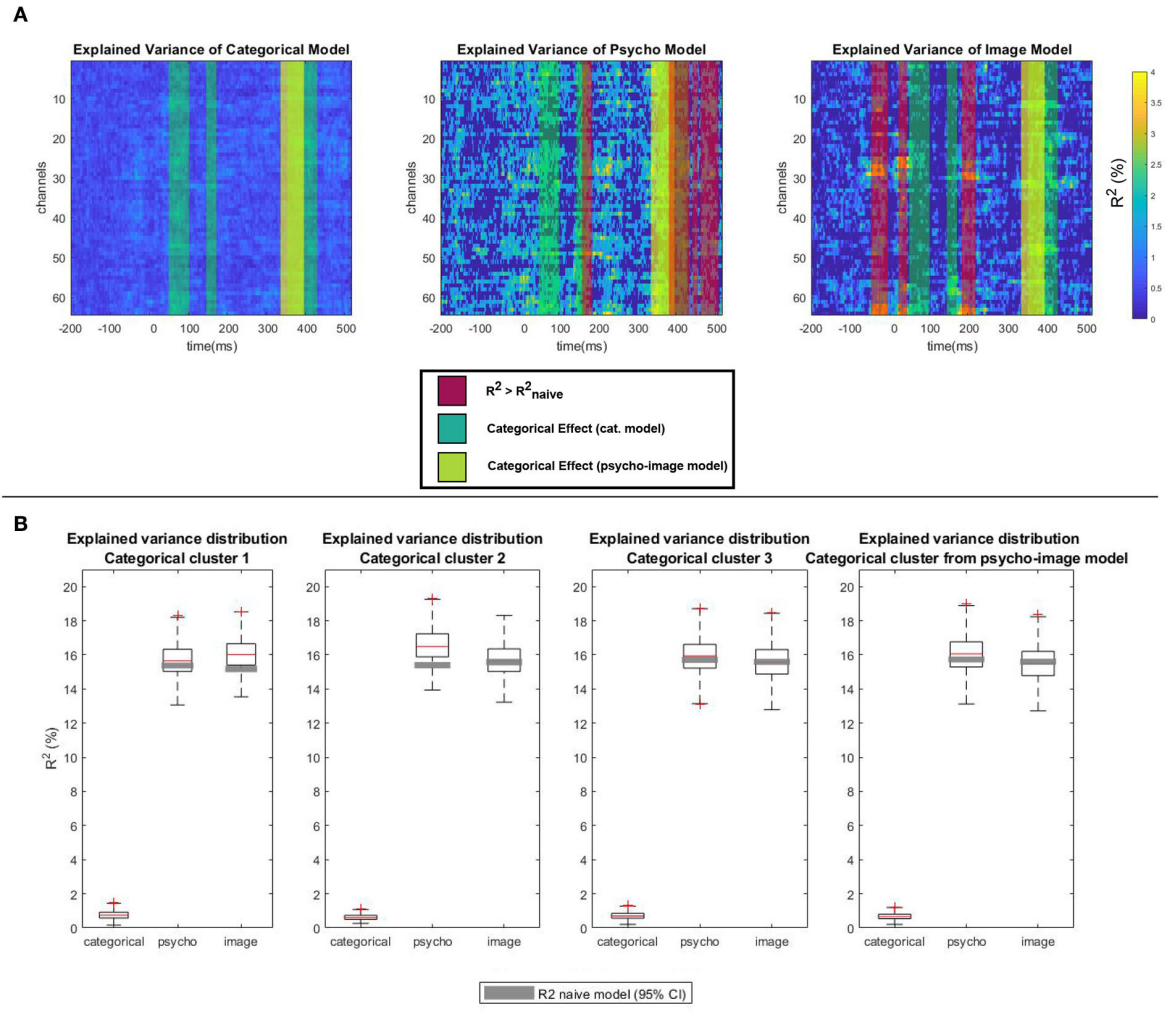
Explained variance quantization. **(A)** Trimmed mean of the explained variance ($R^{2}-R_{naive}^{2}$) across subjects for categorical, psycho, and image models. Regions of significant explained variance (red bands), significant categorical contrast when using the categorical model (dark green bands), and significant categorical contrast when using the psycho-image model (light green band) are highlighted. **(B)** Explained variance distribution for each of the identified categorical clusters. The gray zones within the box plots represent the confidence interval of the variance explained when using the corresponding naive model.

The quantization of the variance that is explained by the different types of covariates was performed by analyzing the *R*^2^ distribution across the spatio-temporal regions of significant contrast identified in Figure 11 using the psycho and image models separately (Figure 12). Figure 12A highlights the regions of significant categorical effect on top of the explained variance maps, with the displayed *R*^2^ values resulting from the difference between the *R*^2^ of the considered model and that of the corresponding naive model. A one sample *t*-test followed by the MCC run on the *R*^2^-values gives us the spatio-temporal regions of the ERP where the variance is significantly explained by the focused type of covariates. For the psycho model, the first significant cluster appears between 151 and 177 ms (max *T*-value 5.61 at 158.9 ms on channel FCz, corrected *p* = 0.01) and the second significant cluster ranges from 319 to 490 ms (max *T*-value 9.01 at 367.9 ms on channel F7, corrected *p* = 0.01). For the image model, the first significant cluster appears between –52 and –9 ms (max *T*-value 4.71 at –22 ms on channel O2, corrected *p* = 0.02), the second significant cluster ranges from 16 to 38 ms (max *T*-value 4.47 at 18.3 ms on channel O2, corrected *p* = 0.01) and the third significant cluster starts at 174 ms and ends at 210 ms (max *T*-value 6.79 at 203.8 ms on channel POz, corrected *p* = 0.02). Comparing the spatio-temporal regions of significant explained variance with the clusters considered of high categorical contrast by the model used allows areas where the classification can be biased by the experimental covariates to be detected. In fact, if a spatio-temporal region whose variance is mainly explained by the covariate values overlaps a cluster of high categorical contrast, an algorithm could use the covariate information to perform the classification instead of the actual categorical information.

To measure this overlapping effect, Figure 12B shows the distribution of the explained variance of each model as well as the confidence interval of the variance explained by the corresponding naive model within each categorical cluster. The explained variance from which the distribution is displayed is computed as the difference between the *R*^2^ of the psycho-image model and the *R*^2^ of the model not concerned, e.g., $R_{psycho}^{2}=R_{psycho-image}^{2}-R_{image}^{2}$. In this way, the part of the variance that is explained by the categorical effect is excluded from the comparison, allowing a focus on the effect of the covariates only. The 95% confidence interval of the corresponding naive *R*^2^-values shows the part of the variance that is explained by the increase of the model dimensionality. The categorical effect is displayed separately to provide a reference point. In the first cluster of significant categorical contrast obtained from the categorical model, the inter-quartile range of the *R*^2^-values from the image model (from 15.41 to 16.65%) stands above the 95% confidence intervals of the *R*^2^-values from the corresponding naive model (14.99 to 15.36%). The same behavior is observed in the second categorical regions of interest (ROIs) (categorical model) for the psycho model with the interquartile range spreading from 15.88 to 17.24% and the confidence interval from the naive model between 15.21 and 15.58%. When focusing on the third categorical cluster (categorical model) or the ROI extracted from the psycho-image model, none of the covariates explain a significant part of the variance as the 95% confidence intervals of the *R*^2^ of both naive models are included in the inter-quartile ranges of the *R*^2^-values from the covariate models.

To validate that the categorical cluster found in the late ERP response can be used to perform a reliable classification between categories, the separability between the categorical and the covariate effects should be proven. As described in Section 2.7, the separability can be evaluated by quantifying the part of *R*^2^ that is lost due to the correlation between the categories and the covariates in the regressed signals. We computed the loss in *R*^2^ using Equation 4 within each categorical cluster separately and by adapting the computations to each model. The results are shown in Table 2. As the part of lost *R*^2^ is significantly lower than the explained variance of the categorical model, the covariate effect can be considered almost orthogonal to the categorical effect, meaning their effects can be easily separated when properly modeled. We can note that negative values in Table 2 are mainly due to the correlation between the psycho-linguistic variables and the image features, knowing that the lost *R*^2^ is computed as the combination of both.

**Table 2:** Ninety-five percent confidence intervals of the explained variance of the categorical model compared with the part of the explained variance that is lost in psycho and image models due to the correlation between the covariates and the categories.

***R*^2^ (%)**	**Cluster 1**	**Cluster 2**	**Cluster 3**	**Cluster 1**
**(95% CI)**	**(cat.model)**	**(cat.model)**	**(cat.model)**	**(psycho**
				**image**
				**model)**
Categorical model	0.72 to 0.82	0.60 to 0.67	0.69 to 0.79	0.66 to 0.75
Psycho model	–0.14 to 0.01	–0.17 to 0.08	–0.18 to 0.04	–0.07 to 0.02
Image model	–0.05 to 0.11	–0.12 to 0.01	–0.18 to 0.06	–0.10 to 0.03

The full analysis of the explained variance highlights the influence of the image features on the regressed ERP around 70 ms and the influence of the psycho-linguistic variables around 150 ms. The late evoked response around 350ms exhibits high independence to the covariate effects, making it a good candidate to perform a reliable classification between living and non-living entities from the EEG trials.

## 4. Discussion

The aim of this research is to inform the experimenter about the uncontrolled factors influencing the neural process of interest. As such, we have investigated how some psycho-linguistic variables and image features impact the EEG signals recorded during a visual priming task with a focus on the represented item origin (natural vs. manufactured). In other words, we have represented the difference between the EEG trials of both categories as a contrast trial and extracted the spatio-temporal areas where the chosen covariates significantly affect the regression process.

When the information on covariates is not modeled by the regression algorithm, any bias on these variables in the dataset, i.e., improper balance in both categories, can be exploited by the BCI algorithm in charge of performing the classification of the EEG trials, leading to the increased probability of misclassification as the algorithm bases its decision on some covariate values instead of the categorical effect itself. This effect increases with the complexity of the model as can be observed in Figure 11 by comparing the clusters wrongly considered as regions of high categorical contrast. In the late evoked response, the cluster of significant contrast is indeed wider when using the naive psycho-image model (26 dimensions) than with the categorical model (3 dimensions). Knowing that all the models used in this study are linear models, the biasing effect could be even worse in BCI applications that use more complex algorithms. As an equal balance of covariate values in the different categories is impossible to reach if we want to keep an acceptable diversity of stimuli in the experiment, a solution is to model the covariate effects as we propose in this study through our hierarchical linear modeling. The modeling solution can be used by neuroscientists to separate category-related from covariate-related responses without balancing covariate values across categories only in the case of demonstrated separability. As we proved in this study, the separation can be done in the case of visual stimulation by natural vs. manufactured items, but this demonstration has to be reiterated for other experimental conditions.

The quantization of the variance explained by each of the covariate types separately, as shown in Figure 12, revealed that both psycho-linguistic variables and image features have an effect on the ERP, but these effects appear outside of the spatio-temporal regions of significant categorical contrast between both categories. A BCI experiment aiming to classify EEG trials into evoked responses induced by natural or manufactured item visual stimulation can therefore take advantage of this finding to perform reliable classification based on the regions of dominant categorical information. Similar to in other BCI experiments, spatio-temporal regions of maximal categorical contrast and minimal covariate effects has first to be identified using rigorous statistical tests, as proposed in the present framework, to state that a reliable classification is possible without requiring specific experimental design considerations. Naturally, this assumption is only valid in the case that the studied covariates are properly chosen and fairly represent the most known potential biasing effects. The discovery of novel stimulus properties impacting the classification will further improve the efficiency of the proposed pipeline.

This research has been led in a reproducible way and the code has been developed using the open-source FieldTrip (Oostenveld et al., 2010) and LIMO EEG (Pernet et al., 2011) toolboxes. Therefore, any other task inducing an evoked response can be analyzed by following the presented method to identify experimental features affecting the distinguishability of the differences in the EEG induced by stimuli of distinct categories. Depending on the type of stimuli and the studied categories, other covariates can be considered in the design of the model to extract their impact on the corresponding ERP. However, as the covariates selection is a critical step of this framework, it should be done carefully by considering the psychological effects related to the experimental task. The presented experimental design being a semantic task, the study of psycho-linguistic features was necessary. The selected variables are those proposed by Alario et al. (2004) who demonstrated their influence on the ERPs related to picture naming tasks. Moreover, as the experiment involves visual stimulation by displaying pictures on a screen, the inner image properties were worth examining. The spatial and spectral features that are traditionally studied such as entropy, energy, and maximal frequency were computed and included in the analysis. Special attention should be paid to the number of selected covariates as more variables could catch a more uncontrolled effect, but decreases the significance between the effect of increased dimensionality and the covariate effect itself. On the contrary, fewer covariates could lead to missing significant covariate effects. The model dimension is also limited by the number of trials available by the subject as performing a regression with more parameters than observations results in overfitting. As the categories are exclusive, the minimum number of trials can be found by multiplying the number of covariates by the number of categories. The number of different subjects will affect the significance of the computed statistical values as these computations are performed at the second level of the hierarchical modeling.

The proposed method should be fully integrated into the design of a BCI experiment by performing a preliminary test with a first set of participants to identify the spatio-temporal regions of significant categorical contrast and evaluate the separability between the covariate and the categorical effects in those regions. In the case of proven separability, the BCI classifier can be trained using the identified regions of interest only. Otherwise, an additional process consisting in balancing the biasing covariates across categories should be done before starting the training procedure.

We draw special attention to the extent of this study. The provided method does not give any insight into internal brain processes responsible for discriminating information coming from specific categories or covariates. It rather aims to unveil the impact of uncontrolled variables on the ability to identify parts of an ERP that exhibits a high contrast between experimental conditions.

The EEG signal being highly complex to interpret and linked to actual brain processes, future work will consist of performing covariate analysis on brain source activity obtained from the recorded EEG signal by source reconstruction techniques. Furthermore, as the proposed framework limits the analysis to the temporal response, the further spectral analysis would allow unknown frequency domain or non-timelocked effects to be revealed.

We hope this research will pave the way to discovering as many experimental biases as possible and optimize the process of designing BCI protocols. In that direction, future study will consist of building a common database with results from different applications. We encourage authors to add their contributions as they go along.

## Data availability statement

The original contributions presented in the study are publicly available. This data can be found at: Zenodo, https://zenodo.org/record/7298746#.Y2kKIXbMK3A, doi: 10.5281/zenodo.6371466.

## Ethics statement

The studies involving human participants were reviewed and approved by Ethics Committee Erasme Hospital. The patients/participants provided their written informed consent to participate in this study.

## Author contributions

LLa and CP designed the proposed method. LLa performed the data analysis. LLa, CP, and VV wrote the paper. EW, AM, and IS designed the experimental task. EW and AM acquired the experimental data. CP, BG, LLe, and LR supervised the research. All authors contributed to the article and approved the submitted version.

## Funding

LLa was funded through a Ph.D. grant from the Fonds pour la Formation à la Recherche dans l'Industrie et l'Agriculture (FRIA), Belgium. EW and AM are funded by the French Community Ministry—general direction of non-mandatory education and scientific research in Belgium through the program of Action de Recherche Concertées (ARC), reference ARC-19/23 UMONS3, Belgium.

## Conflict of interest

The authors declare that the research was conducted in the absence of any commercial or financial relationships that could be construed as a potential conflict of interest.

## Publisher's note

All claims expressed in this article are solely those of the authors and do not necessarily represent those of their affiliated organizations, or those of the publisher, the editors and the reviewers. Any product that may be evaluated in this article, or claim that may be made by its manufacturer, is not guaranteed or endorsed by the publisher.

## Footnotes

^1^The complete description and computation method of each image property are provided in the code available through this repository: https://github.com/numediart/Covariates_Analysis.
